# Medical Treatment of Gastroenteropancreatic Neuroendocrine Tumors

**DOI:** 10.3390/cancers4010113

**Published:** 2012-02-08

**Authors:** Anja Rinke, Patrick Michl, Thomas Gress

**Affiliations:** Department of Gastroenterology, University Hospital Marburg, Baldinger Strasse, Marburg D-35043, Germany; E-Mails: michlp@med.uni-marburg.de (P.M.); gress@med.uni-marburg.de (T.G.)

**Keywords:** neuroendocrine tumor, neuroendocrine carcinoma, somatostatin analogues, interferon-α, mTOR inhibitor, multikinase inhibitor, chemotherapy

## Abstract

Treatment of the clinically and prognostically heterogeneous neuroendocrine neoplasms (NEN) should be based on a multidisciplinary approach, including surgical, interventional, medical and nuclear medicine-based therapeutic options. Medical therapies include somatostatin analogues, interferon-α, mTOR inhibitors, multikinase inhibitors and systemic chemotherapy. For the selection of the appropriate medical treatment the hormonal activity, primary tumor localization, tumor grading and growth behaviour as well as the extent of the disease must be considered. Somatostatin analogues are mainly indicated in hormonally active tumors for symptomatic relief, but antiproliferative effects have also been demonstrated, especially in well-differentiated intestinal NET. The efficacy of everolimus and sunitinib in patients with pancreatic neuroendocrine tumors (pNET) has been demonstrated in large placebo-controlled clinical trials. pNETs are also chemosensitive. Streptozocin-based chemotherapeutic regimens are regarded as current standard of care. Temozolomide in combination with capecitabine is an alternative that has shown promising results that need to be confirmed in larger trials. Currently, no comparative studies and no molecular markers are established that predict the response to medical treatment. Therefore the choice of treatment for each pNET patient is based on individual parameters taking into account the patient’s preference, expected side effects and established response criteria such as proliferation rate and tumor load. Platin-based chemotherapy is still the standard treatment for poorly differentiated neuroendocrine carcinomas. Clearly, there is an unmet need for new systemic treatment options in patients with extrapancreatic neuroendocrine tumors.

## 1. Introduction

Although neuroendocrine tumors (NET) of the gastrointestinal tract are rare neoplasms, their incidence has been increasing over the last decades [1,2,3]. The clinical presentation as well as the course and prognosis of NETs may vary considerably. The new WHO classification categorizes neuroendocrine neoplasms (NEN) of the gastroenteropancreatic tract into well-differentiated neuroendocrine tumors (NET) G1 and G2 and neuroendocrine carcinomas (NEC) [4] (Table 1). Neuroendocrine tumors may present with characteristic hormone-driven syndromes such as the carcinoid syndrome and the Zollinger Ellison syndrome, but may also be functionally inactive. In patients with hormonally inactive tumors diagnosis frequently occurs incidentally during endoscopic or ultrasound examinations for unspecific or unrelated symptoms. NECs are usually hormonally inactive and patients often present with weight loss, weakness or abdominal pain.

**Table 1:** WHO classification of neuroendocrine neoplasms of the gastroenteropancreatic system [4].

WHO 2000	WHO 2010
1. Well-differentiated endocrine tumour (WDET)	1. NET G1 (carcinoid) NET G2 (carcinoid) *
2. Well-differentiated endocrine carcinoma (WDEC)	
3. Poorly differentiated endocrine carinoma/small cell carcinoma (PDEC)	2. NEC G3 large or small cell type
4. Mixed exocrine-endocrine carcinoma (MEEC)	3. Mixed adenoneuroendocrine carcinoma (MANEC)
5. Tumour-like lesions (TLL)	4. Hyperplastic and preneoplastic lesions

* In case that the Ki67 index exceeds 20%, this NET may be labelled G3.

Approximately half of the NEN patients have already developed distant metastases at the time of diagnosis with liver metastases being the predominant localization in most cases. Surgical resection and locoregional treatments of liver metastases are therefore integral parts of the treatment plan in NET patients.

Medical treatment strategies have to consider hormonal activity, localization of the primary tumor, disease extent, growth behavior and prognosis. The proliferation rate is an important predictor of growth behavior and prognosis. The grading system of NEN is based on the immunohistochemical determination of Ki67 as proliferation marker and mitotic counts [5,6]. In patients with G3 tumors (Ki67 > 20%) platin-based chemotherapy is indicated whereas low-proliferating gastrointestinal NET are usually not chemosensitive.

With the advent of novel small molecule inhibitors such as sunitinib and everolimus the therapeutic armamentarium available for G1 and G2 pancreatic NET has substantially broadened. This review provides an overview on the current therapeutic options for NEN.

## 2. Medical Treatment of NEN

### 2.1. Aims of Treatment

Aims of treatment include:

Inhibition of hormone secretion for symptomatic relief in hormonally active tumors;
Improving or maintaining quality of life;
Inhibition of tumor growth;
Prevention of complications (carcinoid crisis, carcinoid heart disease, bleeding, ileus);
Prolongation of survival.

Medical treatment in NEN patients includes somatostatin analogues, proton pump inhibitors in patients with gastrinoma and Zollinger Ellison syndrome (ZES), diazoxid in insulinoma patients, interferon, chemotherapy, small molecules including multikinase inhibitors and mTOR inhibitors. Bisphosphonates may be additionally used as treatment in patients with bone metastases.

In the following we will give an overview of the role of the different medical treatment options in the treatment algorithms for NEN patients.

### 2.2. Somatostatin Analogues

Since the late 80s somatostatin analogues are well established in the treatment of hypersecretion syndromes to achieve symptomatic relief [7,8,9,10]. The effect is mediated by binding to specific somatostatin receptors on the tumor cells. Treatment with somatostatin analogues can therefore be regarded as the first “molecular targeted” treatment in NEN.

Both somatostatin analogues currently available, octreotide and lanreotide, bind to the receptor subtypes 2 and 5 with high affinity. Both drugs have a longer half-life than endogenous somatostatin. Additionally, depot preparations have been developed (octreotide LAR, lanreotide MP and AG) that allow an administration in monthly intervals.

Somatostatin analogues effectively inhibit hormonal secretion and ameliorate flushing and diarrhea in about 75% of patients with carcinoid syndrome (40%–100%) [7]. Perioperative and periinterventional treatment with octreotide can prevent carcinoid crisis [7,11]. Since the risk of carcinoid heart disease is associated with high serotonin levels [12], early treatment with somatostatin analogues may be able to delay or even prevent the development of carcinoid heart disease. Diarrhea in VIPoma patients is also effectively reduced by somatostatin analogues [7]. Somatostatin analogues reduce acid output in gastrinoma patients but proton pump inhibitors are superior in this indication [13,14]. In insulinoma patients hypoglycemia can be reduced in only half of the patients. As somatostatin analogues additionaly inhibit glucagon secretion and thus may worsen hypoglycemia in some patients, somatostatin analogues should not be initiated on an outpatient basis in these patients. Octreotide and lanreotide are regarded equally effective for the symptomatic control of patients with functioning tumors. The only study directly comparing both compounds did not show significant differences [15].

Pasireotide is a novel somatostatin analogue that exhibits higher binding affinity to receptor subtype 1, 3 and 5 than octreotide and lanreotide. Whether this higher binding affinity in particular to receptor subtype 5 results in a therapeutic advantage as previously suggested [16] is under further investigation (ongoing randomized phase III trial comparing pasireotide LAR and octreotide LAR; NCT00690430).

In addition to symptomatic control, somatostatin analogues have been suggested to elicit antiproliferative activity. The proposed mechanisms involve a direct, receptor-mediated antiproliferative effect due to inhibition of the cell cycle and pro-apoptotic activity as well as due to indirect effects including inhibition of the release of growth factors and trophic hormones, inhibition of angiogenesis and modulation of the immune system [7,17].

Several retrospective and small prospective trials reported disease stabilization in about half of the patients (28%–87.5%) treated with somatostatin analogues whereas tumor regression was rarely observed (less than 10%). Table 2 summarizes the studies reporting antiproliferative effects of octreotide and lanreotide [18,19,20,21,22,23,24,25,26,27,28,29,30,31,32,33].

**Table 2:** Summary of studies reporting antiproliferative effects of somatostatin analogues.

First Author and Year	Patients	SSA/Dose	Progression Prior to Treatment	PR(%)	SD(%)	Additional Remarks
Vinik 1989 [18]	14; carcinoid and pNET	Oct sc100 µg–250 µgq 6–12 h	no	20 *	50	* any regression
Öberg 1991 [19]	23 midgut carcinoids	Oct sc 50 µg–100 mgq 12 h	no	28	36	
Saltz 1993 [20]	34 carcinoid and pNET	Oct 150 µg–250 µg t.i.d.	yes	0	50	
Arnold 1996 [21]	103 GEPNET	Oct sc 200 µg–500 µgt.i.d.	in 50%	0	37 ^a^/54 ^b^	^a^ in patients with documented progression ^b^ in patients without documented progression
di Bartolomeo 1996 [22]	58 GEPNET	Oct sc 500 mg–1000 mgt.i.d.	yes	3	47	
Tomassetti 1998 [23]	18 GEPNET	Lan i.m. 30 mg q 10 d	no	0	78	
Wymenga 1999 [24]	55 functioning GEPNET	Lan i.m. 30 mg q 14 d to q 7 d	no	6	81	
Faiss 1999 [25]	30 GEPNET	Lan sc 5000 µg t.i.d.	yes	6.6	37	
Ricci 2000 [26]	15 GEPNET	Oct LAR 20 mg q 28 d	yes	7	40	
Tomassetti 2000 [27]	16 GEPNET	Oct LAR 20 mg q 28 d	no	0	87.5	
Aparicio 2001 [28]	35 GEPNET	Oct sc 100 µg t.i.d. or Lan i.m. 30 mg q 14 d to q 7 d or both	yes	2.9	57.1	
Shojamanesh 2002 [29]	15 gastrinoma	Oct sc or Oct LAR	yes	6	47	
Faiss 2003 [30]	25 GEPNET	Lan sc 1000 µg t.i.d.	yes	4	28	
Bajetta 2006 [31]	30 GEPNET	LAN MP 60 q 21 d	no	3.6	64.3	
	30 GEPNET	LAN AG 120 q 42 d		0	67.9	
Panzuto 2006 [32]	21 pNET	Oct LAR 30 mg q 28 d	yes	0	45	
Rinke 2009 [33]	85 midgut NET	Oct LAR 30 mg q 28 d *versus* placebo	no	2.4 ^a^	67 ^a^	^a^ at 6 months of treatmentPFS 14.5 *versus* 6.0 months

SSA: somatostatin analogue; PR: partial remission; SD: stable disease; pNET: pancreatic neuroendocrine tumor; Oct: octreotide; sc: subcutaneous application; Lan: lanreotide; GEPNET: gastroenteropancreatic neuroendocrine tumor; i.m.: intramuscular application.

In our PROMID trial—a randomized phase III study to compare time to progression (TTP) in patients with metastatic midgut NET randomly assigned to octreotide LAR 30 mg monthly or placebo—we confirmed the antiproliferative efficacy of somatostatin analogues in this patient cohort.

In the octreotide group TTP was significantly increased to 14.3 months as compared to 6 months in the placebo group [33]. This effect was independent of the functional activity, whereas hepatic tumor load was shown to be of prognostic relevance. The greatest benefit was found in patients with a hepatic tumor burden not exceeding 10%. This suggests that an early treatment of patients with midgut NET might be beneficial although a survival advantage is not proven.

Another placebo controlled study with lanreotide AG 120 mg monthly in patients with hormonal inactive intestinal or pancreatic NET (CLARINET trial) has completed recruitment but results are not available yet. This study will provide further information on the role of somatostatin analogue treatment for the inhibition of tumor growth in particular for pNETs.

Currently, somatostatin analogues are only approved for the symptomatic treatment of carcinoid syndrome and functioning pNETs in most countries. Somatostatin analogues are usually well-tolerated. Side effects include abdominal cramps, nausea, diarrhea and flatulence. Less frequently observed side effects comprise cholelithiasis and cholecystitis, hepatitis, pancreatitis, alopecia and diabetes. Rarely prolongation of QT-interval and arrhythmias have been reported [7].

### 2.3. α-Interferon

IFN-α has been used for the treatment of patients with carcinoid syndrome for more than 20 years. IFN-α binds specifically to surface receptors on the tumor cell and thereby reduces hypersecretion resulting in amelioration of carcinoid syndrome in up to 71% of patients [34]. IFN-α is clearly associated with more side effects than somatostatin analogues which therefore remain the treatment of choice in hormonally active tumors.

IFN-α also exerts antiproliferative effects via inhibition of protein synthesis, immunomodulation and inhibition of angiogenesis. A placebo-controlled trial is not available but several phase 2 studies reported a tumor regression in 0–27% (mean 11%) and tumor stabilization in around 40% of the patients. The median duration of tumor response was 12 to 36 months [35].

In two randomized trials the combination therapy of IFN-α and somatostatin analogue (lanreotide in one trial and octreotide in the other) was not superior to somatostatin analogue monotherapy [30,36]. Combination treatment with IFN-α and somatostatin analogues is therefore not indicated as first line treatment but is an option in patients with carcinoid syndrome not sufficiently controlled with somatostatin analogues alone.

Data on pegylated IFN in patients with NEN is very limited [37]. Pegylated IFN has fewer side effects and is better tolerated but has not been approved for NEN.

### 2.4. mTOR Inhibitors

The mammalian target of rapamycin (mTOR) is a serine/threonine kinase that stimulates metabolism, angiogenesis, growth and proliferation in response to growth factors e.g., insulin like growth factor 1 (IGF-1). Activation of this pathway has been shown in several malignancies, including hereditary syndromes that are associated with neuroendocrine tumors. In patients with tuberous sclerosis complex (TSC) 1/2 as well as in patients with neurofibromatosis the causative gene defects result in a loss of natural inhibition of the mTOR pathway [38]. The specific inhibition of mTOR with drugs such as rapamycin or everolimus inhibits cell proliferation of pancreatic endocrine tumor cell lines [39].

In two phase II trials in patients with advanced neuroendocrine tumors of different origins promising results were shown with temsirolimus and everolimus, respectively [40,41].

In another phase II trial, the so called RADIANT1 study, the efficacy of everolimus in patients with metastatic pancreatic NETs (n = 160) who experienced progression on or after chemotherapy was evaluated. Patients already on octreotide treatment were continued with a combination therapy of octreotide and everolimus (stratum 2, n = 45), whereas the majority of patients received everolimus monotherapy (stratum 1, n = 115). More than two thirds of the patients demonstrated tumor stabilization (stratum 1: 67.8%; stratum 2: 80%), whereas partial remissions occurred in less than 10% of the patients (stratum 1: 9.7%; stratum 2: 4.4%) [42].

The efficacy of everolimus in patients with pancreatic NET was confirmed in the RADIANT 3 trial, a large (n = 410) placebo-controlled phase III study. Patients with progressive low-grade or intermediate-grade pancreatic NET were randomly assigned to 10 mg everolimus or placebo. The median progression-free survival (primary endpoint) was 11.4 months (95% CI, 10.8 to 14.8) with everolimus, as compared with 5.4 months (95% CI, 4.3 to 5.6) with placebo (hazard ratio for disease progression or death with everolimus, 0.34; 95% CI, 0.26 to 0.44; *p* < 0.001) [43]. As reported before, the benefit from everolimus in this patient cohort was seen primarily in tumor stabilization or minor tumor shrinkage (stable disease according to RECIST criteria in 73%) whereas the objective response rate was low (5%). Based on the RADIANT 3 data everolimus has been approved for the treatment of patients with pancreatic NET in the United States and Europe.

Another large phase III trial (RADIANT 2) investigated the role of everolimus in patients with progressive NET and a history of carcinoid syndrome. Only half of the 429 enrolled patients had NENs of the small intestine, the remaining patients comprised bronchopulmonary NETs (15% everolimus + octreotide group, 5% placebo + octreotide group), colonic NETs, pNETs and others. Patients received 10 mg everolimus daily + 30 mg octreotide LAR monthly or placebo + 30 mg octreotide LAR monthly, respectively. The primary endpoint was progression free survival (PFS). Based on central imaging assessment the combination of everolimus and octreotide led to a prolongation of PFS of 5.1 months as compared to placebo + octreotide (16.4 *versus* 11.3 months, HR 0.77; *p* = 0.026), but the pre-specified p level of 0.024 was narrowly missed. According to local radiological assessment in the centers, however, the combination showed a similar risk reduction (HR 0.78) and reached statistical significance (*p* = 0.018) [44].

Further investigations are necessary to define the subgroups of patients who benefit from everolimus monotherapy or combination treatments with somatostatin analogues. Everolimus has so far not been approved for the treatment of patients with carcinoid syndrome.

#### Toxicity

The most common toxicity of everolimus was stomatitis (64% of patients) which was mild in most cases (≥grade3: 7%). In 20%–23% of the patients infections were reported, which were ≥grade 3 in 2%–5%. Low grade diarrhea occurred in 27%–34% of the patients. Hematological toxicity was generally mild, but ≥grade 3 thrombocytopenia occurred in 5% of the patients. Everolimus may also induce or worsen hyperglycemia. A non-infectious pneumonitis was reported in 12%–17% (≥grade 3: 2%) and requires special attention and care [43,44]. Adverse events related to the study drug led to discontinuation of treatment in 13% of the patients receiving everolimus in the RADIANT 3 trial.

### 2.5. Multikinase Inhibitors

As neuroendocrine tumors are highly vascularised and express receptors for vascular endothelial growths factors (VEGFR) there is a rationale for treatment with multikinase inihibitors targeting these receptors.

Several substances, including sorafenib, pazopanib and sunitinib have been administered in phase II trials [45,46,47,48,49] (see Table 3).

Sunitinib is an oral multikinase inhibitor with a broad spectrum of targets including VEGFR, PDGFR, c-kit and RET. An international multicenter phase III clinical trial investigated 37.5 mg sunitinib daily *versus* placebo in patients with progressive low to intermediate grade pNET. Recruitment was terminated prematurely after inclusion of half of the planned number of patients (171 of 340). The independent data and safety monitoring committee observed more serious adverse events and deaths in the placebo group as well as a difference in progression-free survival favoring sunitinib in an interim analysis and recommended premature discontinuation of the trial.

Time to progression was significantly longer in the patients treated with sunitinib as compared to placebo (11.4 months *versus* 5.5 months; HR 0.42; *p* < 0.001). Although the majority of patients treated with sunitinib showed minor tumor shrinkage as best morphological result, the objective response rate was only 9.3% [48]. Based on these data, sunitinib was approved for the treatment of advanced pNET in the USA and Europe. The experience in treating extrapancreatic neuroendocrine tumors is limited and thus sunitinib is currently not approved for this indication.

**Table 3:** Overview on studies with targeted therapies in patients with neuroendocrine tumors.

First Author and Year	patients	Number of Patients	Regimen	PD Prior to Treatment	Design	PR	TTP/PFS	Additional Remarks
Hobday 2007 [45], (abstract)	carcinoid	50	sorafenib 400 mg bid	no	phase II	10%	7.8 months	43% grade 3/4 toxicity
	pNET	43					11.9 months	
Yao 2008 [46]	carcinoid	22	octreotide + bevacizumab	no	randomized phase II	18%	95% at week 18	
		22	octreotide + PEGIFN			0%	68% at week 18	
Kulke 2008 [47]	carcinoid	41	sunitinib 37.5 mg	no	phase II	2.4%	10.2 months	
	pNET	66				16.7%	7.7 months	
Raymond 2011 [48]	pNET	171	sunitinib 37.5 mg	yes	randomized phase III, placebo-controlled	9.3%	11.4 months *versus* 5.5 months (placebo)	340 planned patients; survival advantage
Phan 2010 [49], (abstract)	carcinoid	20	octreotide + pazopanib 800 mg	no	phase II	0%	12.7 months	grade 3/4 hypertension 11.7%
	pNET	31				19%	11.7 months	
Duran 2006 [40]	GEPNET	37	temsirolimus	yes	phase II	6%	6 months	
Yao 2008 [41]	carcinoid	30	5–10 mg everolimus + octreotide	no	phase II	17%	63 weeks	trend to better results at 10 mg dose level
	pNET	30				27%	50 weeks	
Yao 2010 [42]	pNET	115	10 mg everolimus	yes	phase II	9.7%	9.7 months	2 strata, no randomization
		45	10 mg everolimus + octreotide			4.4%	17 months	
Yao 2011 [43]	pNET	410	10 mg everolimus *versus* placebo	yes	randomized phase III, placebo-controlled	5%	11.4 months *versus* 5.4 months (placebo)	
Pavel 2010 [44], (abstract)	carcinoid syndrome	429	10 mg everolimus + octreotide *versus* placebo + octreotide	yes	randomized phase III, placebo-controlled		16.4 months *versus* 11.3 months	only 50% intestinal primary, mixed population

PD: progressive disease; PR: partial remission; TTP: time to progression; PFS: progression-free survival; GEPNET: gastroenteropancreatic neuroendocrine tumor; pNET: pancreatic neuroendocrine tumor; PEGIFN: pegylated interferon-α.

#### Toxicity and Quality of Life

The most frequent adverse events in the sunitinib group were diarrhea (59%), nausea (45%), vomiting (34%), asthenia (34%), and fatigue (32%). Severe adverse events included hypertension (10%), neutropenia (12%) hand foot syndrome (6%) and one case of cardiac failure. Global health related quality of life (measured with EORTC QLQ C30) did not differ between patient groups [48].

The place of mTOR inhibitors and sunitinib in the therapeutic algorithm of patients with pNETs still remains to be defined. The majority of patients in both phase III trials received everolimus and sunitinib, respectively, after failure of somatostatin analogues and/or chemotherapy.

### 2.6. Chemotherapy

Chemotherapy is indicated in patients with poorly differentiated NETs regardless of the primary tumor localization. Systemic chemotherapy is also indicated in mixed adenoneuroendocrine carcinomas including goblet cell carcinoids of the appendix.

In patients with well differentiated G1/G2 pNETs chemotherapy is recommended when tumor progression is observed or as first line treatment (without documented progression) in patients with a high tumor load and G2 differentiation. In other foregut G2 tumors (bronchial, thymus, gastric, duodenal NET) chemotherapy is also an option. In contrast, well differentiated intestinal NET should not be treated with chemotherapy due to lack of documented efficacy.

#### 2.6.1. Chemotherapy in G3 Neuroendocrine Carcinoma

In these highly malignant tumors, chemotherapy with cisplatin and etoposide is considered as standard treatment. Reported objective response rates are high (42%–67%) [50,51], but response duration is short (median 8–9 months) and median overall survival does not exceed 19 months. In elderly patients or patients with renal insufficiency cisplatin may be replaced by carboplatin.

Several other regimens which were investigated in G3 neuroendocrine carcinomas including paclitaxel + carboplatin + etoposide [52]; capecitabine + oxaliplatin [53] and carboplatin + vincristin + etoposide [54] were not shown to be superior to the standard cisplatin and etoposide regimen.

A small study in patients with gastric neuroendocrine carcinoma [55] reported promising results (response rate 75%, median survival 22.6 months, n = 12) using the combination cisplatin + irinotecan.

Furthermore, a Scandinavian group reported good results with a temozolomide based chemotherapy as second line treatment in G3 neuroendocrine carcinoma [56].

#### 2.6.2. Chemotherapy in G1/G2 NET of Pancreatic and Other Foregut Origin

Streptozocin in combination with 5-fluorouracil (5-FU) or/and doxorubicin is the standard regimen in this patient group. Older studies reporting response rates of up to 69% including the use of clinical parameters for the assessment of tumor response [57,58]. In newer studies using established imaging criteria the response rates do not exceed 40%. However, this is still superior to the results of other treatment options including targeted therapies. The duration of response often is long-lasting (time to progression 7–20 months) [59,60,61,62,63].

The main toxicity of streptozocin is renal dysfunction in more than 20% of treated patients including proteinuria and renal failure. Renal function including determination of glomerular filtration rate and proteinuria should therefore be monitored closely in streptozocin-treated patients. Other side effects include nausea/vomiting, impaired glucose tolerance and mild bone marrow toxicity.

Dacarbazine (DTIC) alone or in combination with epirubicin and 5-FU is an alternative regimen with ORR of around 30% [64,65,66]. Monotherapy of DTIC (650 mg/m^2^–850 mg/m^2^ every 4 weeks) can easily be applied in an outpatient setting. Main toxicities are nausea/vomiting and bone marrow suppression.

Temozolomide is an oral chemotherapeutic drug sharing the active metabolite metozolomide with DTIC. Small studies report promising results as monotherapy or in combination with bevacizumab, thalidomide and capecitabine [67,68,69]. The combination of temozolomide and capecitabine as first line treatment in pNET resulted in an impressive response rate of 70% and a 2 year survival rate of 92% [69]. However, this was a retrospective study with a limited number of patients (n = 30) that should be confirmed in a larger prospective clinical trial.

### 2.7. Ongoing Trials and Future Perspectives

Further targeted treatments that are currently investigated include atiprimod (oral STAT3 and AKT inhibitor), cabozantinib (oral multikinase inhibitor), drugs targeting the insulin like growth factor receptor-1 (IGF-1-R) (cixutumumab, MK0646, AMG 479), pazopanib (oral multikinase inhibitor), axitinib (angiogenesis inhibitor, oral multikinase inhibitor), thalidomide, selective and non-selective PI3K inhibitors and the proteasome inhibitor bortezomib.

The efficacy of sunitinib in patients with poorly differentiated neuroendocrine tumors is evaluated in a phase II study.

An ongoing phase III trial compares bevacizumab and octreotide *versus* interferon-α2b and octreotide in patients with progressive carcinoid tumors. Another placebo-controlled phase III trial of everolimus in patients with carcinoid tumors is planned (RADIANT-4) to further evaluate the role of mTOR inhibition in this patient population.

New substances that might be interesting for treatment of NEN comprise inhibitors of heat shock protein 90, inhibitors of the src pathway and hedgehog inhibitors [70,71,72].

Blocking a single signaling pathway in tumor cells often leads to the development of escape mechanisms. This phenomenon could for example be demonstrated in intestinal neuroendocrine tumor cell lines treated with the mTOR inhibitor everolimus [73]. Therefore combination treatments may be more effective in NEN patients. A combinatorial approach can include two targeted treatments (e.g., EGF-R inhibition + mTOR inhibition [74] or IGF-1-R inhibition + mTOR inhibition) but also the combination of one targeted treatment and locoregional treatments (e.g., hepatic artery embolization followed by sunitinib or everolimus; SIRT in combination with sunitinib or everolimus) and the combination of chemotherapy and targeted treatments. In trials with combination treatments a focus on side effects and quality of life is essential.

We clearly need prospective comparative phase III trials of the different therapeutic modalities in pNET patients to evaluate superiority regarding efficacy, tolerance and quality of life. In the future, a better understanding of the different tumor biology and analysis of molecular mechanisms will help to provide a basis for individualized treatment.

## 3. Conclusions

With the advent of novel small molecule inhibitors such as sunitinib and everolimus the therapeutic armamentarium available for G1 and G2 pancreatic NET has substantially broadened. The place of mTOR inhibitors and sunitinib in the therapeutic algorithm of patients with pNETs in respect to presently used therapies such as PPRT or somatostatin analogues still remains to be defined. There is an unmet need for new systemic treatment options in patients with extrapancreatic neuroendocrine tumors.
